# Comparison of prognostic factors in patients in phase I trials of cytotoxic drugs *vs* new noncytotoxic agents

**DOI:** 10.1038/sj.bjc.6601218

**Published:** 2003-09-30

**Authors:** C Han, J P Braybrooke, G Deplanque, M Taylor, D Mackintosh, K Kaur, K Samouri, T S Ganesan, A L Harris, D C Talbot

**Affiliations:** 1 Cancer Research UK Medical Oncology Unit, Churchill Hospital, Headington, Oxford OX3 7LJ, UK

**Keywords:** phase one, cytotoxic, noncytotoxic, prognosis, multivariate analysis

## Abstract

The aims of this study were to identify prognostic variables for toxicity and survival in patients with cancer participating in phase I clinical trials and compare characteristics of those treated with cytotoxic chemotherapy (CT) and non-cytotoxic drugs (non-CT). Data were collected from 420 (114 CT, 306 non-CT) patients enrolled in 16 phase I trials (five CT and 11 non-CT trials) in one cancer centre. Analyses of all patients were used to compare treatment groups, identify predictive variables for toxicity and to estimate prognostic factors in overall survival (OS). These were used to develop a prognostic index (PI). Multivariate analysis found those patients with better performance status, fewer sites of metastases, baseline Hb>12 g dl^−1^ and WBC or LDH in the normal range had significantly better OS. Male gender, platelet count <450 × 10^9^ l^−1^, high WBC or treatment with a non-CT phase I agent significantly reduced the chance of grade 3/4 toxicity. Overall survival was not significantly different between the CT and non-CT groups (260 *vs* 192 days, *P*=0.47) except for those with liver metastases (228 *vs* 137 days, *P*=0.02). Overall tumour response was 4.9% (95% CI: 2.7–7.0%). The PI identified three distinct patient groups with median survival of 321, 257 and 117 days. In conclusion, entry into a phase I trial of a non-CT drug is a safe option for heavily pretreated patients with cancer. The PI generated from these data can estimate the survival probability for patients entering phase I studies.

Careful phase I clinical trial design includes avoidance of unacceptable toxicity to participating patients with cancer and minimisation of the number of patients treated with ineffective drug doses. Despite the need for the development of new therapies, and the importance attached to the findings of phase I trials in oncology, surprisingly little information is published about patient selection and potential prognostic indices that may aid the clinician in predicting, and discussing, the likely outcome for an individual. Increasingly, with the development of biological therapies there is a need to evaluate patient outcome in phase I studies with newer agents in comparison to those treated with traditional cytotoxic drugs. This is important for determining whether or not these trials can be considered as an ethical treatment option or whether they compromise survival and quality of life.

Typically, in oncology, phase I trials have been offered to patients with cancer who have a good performance status and have either failed standard treatment or for whom no standard therapy exists. Despite the emphasis on evaluation of side effects, and determination of a maximum tolerated dose (MTD), tumour responses remain an important secondary end point. Reported response rates for phase I trials are generally between 1 and 10% (Estey *et al*, 1986; Decoster *et al*, 1990; Bachelot *et al*, 2000) with most responses seen at 80–120% of the recommended phase II dose (Von Hoff and Turner, 1991). Cytotoxic drugs that do not show antitumour activity in phase I trials rarely undergo further evaluation (Von Hoff and Turner, 1991; American Society of Clinical Oncology, 1997; Sekine *et al*, 2002).

Previous reviews of phase 1 study data have used multivariable analyses to explore factors associated with toxicity and prognosis. Dosage level and age over 65 years are independently associated with grade 3 or 4 toxicity (Bachelot *et al*, 2000). Poor performance status (WHO grade 2 or 3) (Janisch *et al*, 1994; Bachelot *et al*, 2000), elevated lactate dehydrogenase (Bachelot *et al*, 2000), lower albumin, elevated platelet counts and previous cisplatin therapy (Janisch *et al*, 1994) have all been identified as independent adverse prognostic variables. Analyses have mostly been limited by small sample sizes and have not compared cytotoxic (CT) with non-cytotoxic (non-CT) drugs. For new non-CT agents such as antiangiogenic drugs or matrix metalloproteinase inhibitors, tumour shrinkage may be less likely and chronic drug administration might be required before side effects become apparent. Careful patient selection is therefore important to maximise the information obtained from these clinical trials.

In this retrospective study, we compared patients from a single centre entered into phase I studies with CT drugs or non-CT drugs. A range of clinical, biochemical and haematological factors were assessed by both univariate and multivariate analysis for toxicity and to provide prognostic indicators that could be used for development of a predictive model for survival in patients entered into phase I studies.

## METHODS

### Patients

We identified 16 (CT drugs: five; non-CT drugs: 11) phase I clinical trials conducted by the Cancer Research UK Medical Oncology Unit, Oxford, between 1991 and 2000 (Table 1

**Table 1:** Phase I trials included in analysis

**Investigational agent/molecular target**	**Number of patients**	**Reference**
*Non-cytotoxic*		
Matrix metalloproteinase inhibitor	9	In preparation
Matrix metalloproteinase inhibitor	6	Macaulay *et al* (1999)
Mitochondrial inhibitor	11	Propper *et al* (1999)
Matrix metallo-proteinase inhibitor	92	Levitt *et al* (2001)
8-Chloro cyclic AMP	33	Propper *et al* (1999)
Antiangiogenesis	50	Jones *et al* (1999)
Dendritic cell therapy	5	Chao *et al* (2003)
Gene therapy	23	Unpublished
EGFR inhibitor	5	Twelves *et al* (2002)
Protein kinase C partial agonist	56	Philip *et al* (1993)
Retinoid	16	Jones *et al* (2003)
		
*Cytotoxic*		
Topoisomerase I/II inhibitor	12	Propper *et al* (2003)
Topoisomerase I inhibitor	35	Braybrooke *et al* (2003)
Topoisomerase I inhibitor	21	Submitted for publication
Bio-modulation of 5-FU	21	Braybrooke *et al* (2000)
Cyclophosphamide, methotrexate and infusional 5-FU	25	O'Byrne *et al* (1998)

). A total of 420 individual patient records and case report forms were used as the data source. Information collected included demography, performance status, diagnosis, stage, number/sites of metastases, previous therapy, haematological and biochemical indices, start/end date of study drug treatment, dose level, toxicity grades, date last seen and date of death. Tumour response was assessed by WHO (Miller *et al*, 1981), or South West Oncology Group (SWOG) criteria (Green and Weiss, 1992) according to the phase I study protocol. Toxicity was assessed by the NCI-CTC criteria (National Cancer Institute: Guidelines, 1988). When required by the individual trial protocol, radiological review was performed by an independent review panel. In other cases, tumour response was reported by an independent consultant radiologist blinded to the treatment intervention. All studies were conducted in accordance with the Declaration of Helsinki and were approved by Cancer Research UK and the local research ethics committee.

### Statistical methods

Contingency tables were analysed using the Pearson's *χ*^2^ test. The survival was measured from the first day of treatment on the phase I trial to the time of death or the time of last follow-up. The log-rank test was used to perform univariate analysis for survival and the survival curves were estimated by the Kaplan–Meier method. Prognostic factors for survival were evaluated in multivariate analyses by Cox proportional hazards regression. Logistic regression was performed for estimating the predictors of grade 3/4 toxicity in multifactorial analysis. All statistics were performed using the Stata package release 7.0 (Stata Corporation, TX, USA). Based on the five risk factors from the multivariate survival model, we generated a prognostic index as a survival probability estimator (*S*(*t*)=exp[-*H*_0_(*t*) × exp(PI)], where *S*(*t*)=survival time, *H*_0_=a step function over time, *t*=time, and PI=prognostic index. The hazard and estimated survival probability at any time depends only upon the PI.

## RESULTS

### Patients

A total of 420 patients (210 males, 210 females), median age 56 years (range 22–87 years) were included in the analysis. The cancer types reflect referral patterns to the Oxford Medical Oncology Unit, with the majority of patients having colorectal (78), melanoma (65), breast (45), renal (39), ovarian (38) and lung (37) cancers as their primary tumour site. The overall response rate for all patients was 4.9% (0.3% complete response, 4.6% partial response) with 19.2% patients obtaining disease stabilisation for 3 months or longer.

### Comparison of CT and non-CT groups

In all, 306 patients were treated with non-CT drugs and 114 with CT (Table 2

**Table 2:** Comparisons between CT and non-CT groups

**Variable**		**Non-cytotoxic**	**Cytotoxic**	***P*-value (test)**
Number of patients		306	114	
Female	Total 210	131 (42.8%)	79 (69.3%)	<0.001
Male	Total 210	175 (57.2%)	35 (30.7%)	(*χ*^2^)
				
Median age (range)		58 (22–87)	55 (26–76)	0.04 (M–W)
WHO performance status				
0		143 (48.2%)	35 (31.5%)	
1		118 (39.7%)	61 (55.0%)	0.02
2		33 (11.1%)	14 (12.6%)	(Fisher's)
3		3 (1.0%)	1 (0.9%)	
*Number of metastatic sites*				
0–1		142 (48.6%)	47 (41.6%)	0.20
⩾2		150 (51.4%)	66 (58.4%)	(*χ*^2^)
*Lung involvement*				
No		221 (73.7%)	75 (65.8%)	0.11
Yes		79 (26.3%)	39 (34.2%)	(*χ*^2^)
*Liver involvement*				
No		228 (75.0%)	63 (55.3%)	<0.001
Yes		76 (25.0%)	51 (44.7%)	(*χ*^2^)
Median overall survival, days (range)		192 (4–2405)	260 (10–1136)	0.47 (LR)
Number of deaths at 3 months (%)		63 (20.6%)	18 (16%)	
*Median overall survival (days)*				
Liver involvement				
No		235	260	0.86 (LR)
Yes		137	228	0.02 (LR)
Lung involvement				
No		202	271	0.40 (LR)
Yes		162	228	0.83 (LR)
Median duration of trial (days)		51	73	< 0.001 (M–W)
Response to treatment: (overall)				
CR (0.3%)		0	1 (0.9%)	
PR (4.6%)		3 (1.0%)	15 (13.2%)	< 0.001
SD (19.2%)		42 (13.7%)	36 (31.6%)	excluding NE
PD (64.8%)		221 (72.2%)	55 (48.2%)	(Fisher's)
NE (11.1%)		40 (13.1%)	7 (6.1%)	
*Lactate dehydrogenase*				
normal range		165 (56.1%)	67 (75.3%)	0.001
>normal range		129 (43.9%)	22 (24.7%)	(*χ*^2^)

M–W=Mann–Whitney *U*-test; Fisher's=Fisher's exact *t*-test; LR=log-rank test; *χ*^2^=chi-squared test; CR=complete response; PR=partial response; SD=stable disease; PD=progressive disease; NE=not evaluable. All responses assessed using standard WHO criteria.

). The median age was slightly lower for patients receiving CT drugs (55 *vs* 58 years; *P*=0.04, Mann–Whitney *U*-test), although more patients had a performance status of zero in the non-CT trials (*P*=0.02, Fisher's exact test). Significantly, more women were treated with CT drugs (*P*<0.001, *χ*^2^ test) reflecting inclusion of a study of a novel schedule of cyclophosphamide, methotrexate and infusional 5-FU for women with breast cancer (O'Byrne *et al*, 1998). There was no significant difference in the numbers of sites of metastases between groups (*P*=0.2, *χ*^2^ test) but significantly more patients in the CT studies had liver metastases (*P*<0.001, *χ*^2^ test). Objective tumour response rates were higher in patients receiving CT therapy (*P*<0.001, *χ*^2^ test). The median duration of time on trial was shorter for patients receiving non-CT treatment (51 *vs* 73 days, *P*<0.001, Mann-Whitney *U*-test). There was no significant difference in the median overall survival (192 days (range 4–2405), non-CT *vs* 260 days (range 10–1136), CT; *P*=0.47, Log-rank test) and no difference in the numbers of patients who had died within 3 months of study start (20.6% non-CT *vs* 16% CT). However, in patients with liver metastases (*n*=127), treatment with CT drugs resulted in a significant survival advantage compared to non-CT drugs (median 228 *vs* 137 days, *P*=0.02, log-rank test). There was no difference in survival between groups for patients with lung metastases.

### Survival analysis

When all patients were analysed together by univariate analyses, WHO performance status>1, white blood count above the normal range, low haemoglobin (<12 g dl^−1^), raised platelets (>450 × 10^9^ l^−1^), lactate dehydrogenase above the normal range, low albumin, number of metastatic sites>1, presence of liver and/or lung metastases and stage of disease were all significant adverse factors for predicting reduced survival (Table 3

**Table 3:** Univariate survival analysis, all patients

**Variable**		**Median OS (days)**	**No. of patients**	**Event (deaths)**	**Log-rank, *P*-value**
*Performance status*: (WHO)					
0		292	178	125	<0.001
⩾1		154	230	165	
*White blood count*					
Normal range		228	364	256	<0.001
>Normal range		120	51	37	
*Haemoglobin*					
⩽12 g dl^−1^		154	195	147	<0.001
>12 g dl^−1^		293	220	220	
Lactate dehydrogenase	Normal range	281	232	169	<0.001
	>Normal range	137	151	106	
*Platelets*					
⩽450 × 10^9^ l^−1^		246	362	250	<0.001
>450 × 10^9^ l^−1^		105	55	43	
*Stage*					
	2 or 3	468	22	16	0.01
	4	193	375	272	
*Lung/liver involved*	No	249	207	125	0.03
	Yes	189	207	165	
*Albumin*					
	Low	162	173	120	<0.001
	High	249	229	159	
*Drug*	Non-cytotoxic	192	306	216	0.47
	Cytotoxic	260	114	80	
*Number of sites of metastases*	0–1	264	189	116	0.003
	⩾2	186	216	167	

). Tumour type and treatment with either CT or non-CT drugs were not significant predictors of survival. Data on serial weight change were not available from the majority of studies and could not be included in the analysis. In multivariate analysis, only five factors remained as independent prognostic variables–performance status, white blood count, haemoglobin, lactate dehydrogenase and the number of metastatic sites (Table 4

**Table 4:** Multivariate survival analysis, all patients

**Variable**	**Hazard ratio**	**95% CI**	***P*-value**
*Performance Status (WHO)*			
0	Baseline		
⩾1	1.48	1.15–1.92	0.003
*White blood count*			
normal range	Baseline		
above normal range	1.65	1.13–2.40	0.01
*Haemoglobin*			
>12 g dl^−1^	Baseline		
⩽12 g dl^−1^	1.60	1.23–2.08	<0.001
*Lactate dehydrogenase*			
Normal range	Baseline		
>Normal range	1.54	1.18–2.01	0.002
*Number of sites of metastases*			
0–1	Baseline		
⩾2	1.5	1.16–1.93	0.002

). These factors were used for development of a prognostic index as a predictive model for survival (see Discussion).

### Analysis of toxicity

In univariate analyses for grade 3 or 4 toxicity, treatment with CT or non-CT drugs, gender, performance status and baseline creatinine, albumin and age were all significant factors (Table 5

**Table 5:** Univariate analysis for WHO grade 3/4 toxicity, all patients

**Variable**	**Odds ratio**	**Toxicity grade <3**	**Toxicity grade 3/4**	***P*-value**
*Treatment*				
Non-cytotoxic drugs	Baseline	191	107	<0.001
Cytotoxic drugs	2.95	43	71	
*Sex*				
Female	Baseline	94	111	<0.001
Male	0.41	140	67	
*Performance status (WHO)*				
0	Baseline	114	64	0.01
⩾1	1.69	115	109	
*Creatinine (range)*				
Lower	Baseline	64	74	
Middle	0.62	81	58	0.006
Upper	0.46	87	46	
*Age (Years)*				
<65	Baseline	163	141	0.03
⩾65	0.6	71	37	
*Albumin*				
Normal range	Baseline	87	83	0.03
>Normal range	0.63	141	85	

). Haematological indices, lactate dehydrogenase, dose level and disease stage were not significant. In multivariate analysis, the class of drug, elevated platelet count, low white blood count and gender were the only significant independent variables for toxicity (Table 6

**Table 6:** Multivariate analysis for WHO toxicity grade 3/4

**Variable**	**Hazard ratio**	**95% CI**	***P*-value**
*Drug*			
Non-cytotoxic	Baseline		
Cytotoxic	2.65	1.66–4.24	<0.001
*Platelet*			
⩽450 × 10^9^ l^−1^	Baseline		
>450 × 10^9^ l^−1^	2.17	1.14–4.13	0.02
*Sex*			
Female	Baseline		
Male	0.48	0.31–0.73	<0.001
*White blood count*			
Low	Baseline		
Middle	0.66	0.40–1.10	0.11
High	0.52	0.31–0.89	0.02

).

## DISCUSSION

The continued development of new anticancer drugs is dependent upon patient entry into phase I clinical trials. Treating physicians have an ethical and moral responsibility to be sure that they do not compromise patient survival or expose patients to unacceptable toxicity. Equally, while not the primary end point of phase I studies, individual trials have limited statistical powers and potentially beneficial therapies should not be rejected without thorough assessment. This retrospective analysis of 420 patients, treated in 16 phase I studies, is the first to address whether there are differences in outcome for patients entered into CT and non-CT phase I trials. Conventional multivariate analyses were used to elucidate which factors appear to be important for predicting toxicity and survival. The extent to which physician bias may have influenced patient entry into studies has not been determined in the analysis but must be considered. All patients were treated at a single institution, by three physicians, using agreed shared protocols to minimise bias.

Not surprisingly, treatment with a CT drug as opposed to a non-CT agent was an independent variable for predicting grade 3 or 4 toxicity. Traditional phase I design includes dose escalations, by cohort, until significant toxicities are observed. With studies of non-CT agents, conventional criteria for achieving a maximum tolerated dose are not always achieved. This may account for the lack of significance of dose level for predicting grade 3/4 toxicity. Other factors that did predict toxicity included a low white blood count, platelet count above the normal range and female sex. Haematological indices provide nonspecific markers of inflammatory changes and thus may be expected to predict for toxicity. A low white blood count may also reflect extensive prior cytotoxic treatment or an immunocompromised patient. Gender was an unexpected predictor. A report of patients with colorectal cancer found greater 5-fluorouracil (5-FU)-induced toxicity in women (Sloan *et al*, 2002). In our analysis, data from a phase I study of cyclophosphamide, methotrexate and infusional 5-FU in breast cancer patients was included. However, if this study is excluded the toxicity results remain significant. Female gender is also a significant factor for toxicity in non-CT trials. This may be due to patients with breast or ovarian cancer entering phase I trials after extensive previous cytotoxic chemotherapy and radiotherapy. The large number of trials of different agents, with multiple mechanisms of action, suggest to us that physiological factors are unlikely to account for the increased grade 3/4 toxicity in females although this cannot be ruled out.

One of the aims of this analysis was to determine if there is a difference in outcome between CT and non-CT phase I trials. Direct comparison from data in this analysis has been restricted by the imbalance in number of patients receiving CT *vs* non-CT trials (114 *vs* 306 patients respectively). Initial observations on patient demographics found significantly more women and patients with liver metastases received CT drugs while more patients with an initial performance status of zero were entered into studies with non-CT drugs. These differences may indicate that oncologists are selective about which trials are offered to patients. Alternatively, patients considering entry into a phase I trial are selective about which drugs they prefer to receive, based on perceived efficacy or toxicity. A previous study on perceptions of patients entering phase I trials suggested that many were strongly motivated by the hope of therapeutic benefit (Daugherty *et al*, 1995) and, if offered a choice of studies, may consider this carefully.

Tumour response and survival were secondary end points in every phase I trial conducted. In this study, more patients obtained an objective tumour response and disease stabilisation when treated with a CT drug compared to a non-CT drug (14.1% *vs* 1.0% and 31.6% *vs* 13.7%, respectively). The high response rates seen with CT drugs reflects the inclusion of two phase I trials of novel schedules of drugs known to have significant antitumour activity (i.e. cyclophosphamide, methotrexate and infusional 5-FU; 5-FU, folinic acid and interferon). Subanalysis of response rates in the phase I studies of novel CT drugs, excluding these two trials, still showed a partial response rate of 10.3%, which is higher than many previous reports (Estey *et al*, 1986; Decoster *et al*, 1990; Bachelot *et al*, 2000; Sekine *et al*, 2002). Several of these drugs were new selective topoisomerase inhibitors and would be predicted to have antitumour activity. Tumour type was not a significant predictor of outcome in this analysis, although numbers of patients with each cancer site were small. It is not clear why patients in phase I studies of CT drugs remained on trial medication for significantly longer (73 *vs* 51 days). Disease progression was the usual reason for discontinuing treatment. Disease response and stabilisation favoured patients receiving CT rather than non-CT drugs. It is possible that patients treated with cytotoxic agents stayed on study longer because they responded or had stable disease. While higher response rates were seen with CT drugs, there was no statistically significant difference in patient survival between the classes of drugs (260 *vs* 192 days, *P*=0.47). This finding should be interpreted with caution. More patients with a performance status of zero entered studies with non-CT drugs (48.2% of patients *vs* 31.5% of patients in CT trials, *P*=0.02) and survival may, therefore, reflect the natural history of the cancer rather than an effect of treatment. However, when groups were matched for performance status there was still no significant difference (*P*=0.45), although numbers of patients were small. Analysis of patients with liver metastases found that those treated with a non-CT drug had poorer survival compared to those treated with a CT drug (137 *vs* 228 days, *P*<0.02). This may be of importance when considering the choice of phase I study although must be considered with caution, as only 127 patients were included with liver metastases. The extent of liver disease was not known.

No comparable data set exists for survival of patients who elected not to enter phase I trials. Thus, assessment of how entry into phase I trials affects survival in this patient population cannot be made. It would seem unlikely, based on the toxicity data, that participation in a phase I clinical trial compromised survival. The impact on quality of life is not known.

Establishing independent prognostic indices for survival of patients treated in phase 1 studies makes it possible to develop a predictive model for the likely survival of individual patients. The five factors we identified in this analysis were: performance status (zero *vs* one or greater), white blood count (normal range *vs* above normal range), haemoglobin (less than or equal to 12 g dl^−1^ *vs* greater than 12 g dl^−1^), lactate dehydrogenase (normal range *vs* above normal range) and number of sites of metastases (zero or one *vs* two or more sites). These risk factors can be used to identify whether patients have a good, intermediate or poor risk of survival (Table 7

**Table 7:** Prognostic model for prediction of survival

***Formula***				
***S*(*t*)=exp[-*H*_0_(*t*) × exp(PI)]**				
***S*(*t*)**=suvival probability at time t				
***H*_0_**=a step function over time				
**PI**=prognostic index				
				
**Group**	**Risk factors (*n*)**	**Patients (*n*)**	**Events (*n*)**	**Median survival (days)**
Good risk	0–1	136	91	321
Intermediate risk	2	96	64	257
Poor risk	⩾3	127	105	117
				
**Log-rank test:**				
	Overall:		*P*< 0.001	
	Good *vs* Intermediate		*P*=0.004	
	Intermediate *vs* poor		*P* <0.001	
	Good *vs* poor		*P*<0.001	

Patients were divided into three groups depending upon the number of adverse risk factors (i.e performance status>1, white blood count>normal range, haemoglobin <12 g dl^−1^, lactate dehydrogenase above normal range, number of sites of metastases>2). Each risk factor counts as 1.

, Figure 1

**Figure 1:**
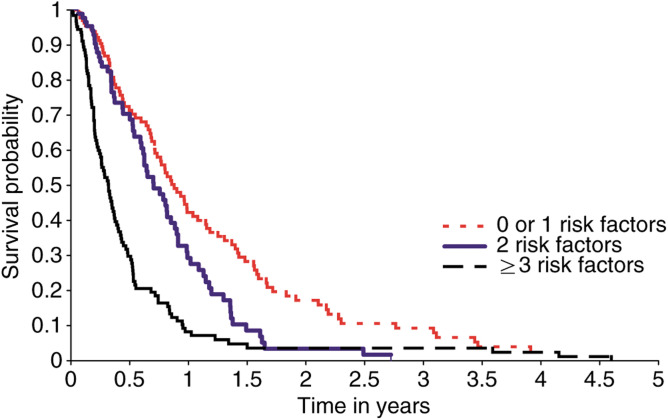
Predicted survival for patients entered into phase I clinical trials using independent prognostic indices identified from multivariate analysis (performance status, white blood count, haemoglobin, lactate dehydrogenase and number metastastic sites). Patients are categorised into good (0 or 1 risk factors), intermediate (2 risk factors) or poor risk (⩾3 risk factors).

). Independent analysis shows a significant difference between each group for survival, ranging from a median of 321 days for the good risk group to 117 days for the poor risk group. Prognostic factors for individual patients can be entered into this model and median survival estimated.

Of the 16 phase I studies included in uni- and multivariable analyses, two studies employed ‘standard’ chemotherapy administered in a novel schedule or in combination with biological modulators. These potentially more effective treatments might therefore have biased the results of this study. Exclusion of these two studies, from the comparisons of CT and non-CT phase I trials, univariables and multivariable analyses did not significantly affect the statistical results.

The development of prognostic indices for toxicity and survival are important considerations in the design of early phase clinical trials. This retrospective analysis is the first to be reported, that directly considers phase I non-CT trials. Differences were identified in baseline characteristics of patients entered into non-CT phase I studies compared to CT trials. It is not clear whether this is due to physician bias or patient choice. Most importantly, no significant difference in median overall survival was found between the two treatment groups. The use of prognostic models, as proposed from this analysis, may be important for discussing patient entry into trials and could, in future, be helpful as part of the inclusion criteria. The model developed in this study uses five independent factors and further work, with other large data sets from phase 1 trial centres, is required for validation and determination of its use prospectively.
